# Direct Toll-Like Receptor 8 signaling increases the functional avidity of human CD8+ T lymphocytes generated for adoptive T cell therapy strategies

**DOI:** 10.1002/iid3.43

**Published:** 2015-02-19

**Authors:** Jean-François Chatillon, Mohamad Hamieh, Florence Bayeux, Claire Abasq, Emilie Fauquembergue, Aurélie Drouet, Florian Guisier, Jean-Baptiste Latouche, Philippe Musette

**Affiliations:** 1 University of RouenRouen, France; 2 Institut National de la Santé et de la Recherche Médicale (INSERM) U905Rouen, France; 3 INSERM U1079Rouen, France; 4 Rouen University HospitalRouen, France

**Keywords:** cytotoxic T lymphocytes, functional avidity, immunotherapy, melanoma, Toll-Like receptors

## Abstract

Adoptive transfer of *in vitro* activated and expanded antigen-specific cytotoxic T lymphocytes (CTLs) is a promising therapeutic strategy for infectious diseases and cancers. Obtaining *in vitro* a sufficient amount of highly specific cytotoxic cells and capable of retaining cytotoxic activity *in vivo* remains problematic. We studied the role of Toll-Like Receptor-8 (TLR8) engagement on peripheral CTLs activated with melanoma antigen MART-1-expressing artificial antigen-presenting cells (AAPCs). After a 3-week co-culture, 3–27% of specific CTLs were consistently obtained. CTLs expressed TLR8 in the intracellular compartment and at the cell surface. Specific CTLs activated with a TLR8 agonist (CL075) 24 h before the end of the culture displayed neither any change in their production levels of molecules involved in cytotoxicity (IFN-γ, Granzyme B, and TNF-α) nor major significant change in their cell surface phenotype. However, these TLR8-stimulated lymphocytes displayed increased cytotoxic activity against specific peptide-pulsed target cells related to an increase in specific anti-melanoma CTL functional avidity. TLR8 engagement on CTLs could, therefore, be useful in different immunotherapy strategies.

## Introduction

The Toll-like receptor (TLR) family, which is constituted of 12 and 10 functional receptors in mice and humans, respectively 1, has been described in almost every tissue and different species 2,3. TLRs are known to be involved in innate immunity. It is now clear that different TLR ligands can play a key role on antigen-presenting cells (APC), such as dendritic cells by maturing them into fully potent ones with strong co-stimulatory, secreting capacities and stabilized peptides in the groove of major histocompatibility complex molecules 4. Recently, the idea that these innate immune receptors could play a direct role on adaptive immune cells was investigated.

In total T cells, TLR engagement has been mainly shown to be involved in IFNγ secretion 5,6.

In CD4+ T cells, TLRs have been shown to play a key role in effector cell proliferation and survival, T helper subset differentiation, and regulatory T cell function reduction 6.

In murine CD8+ T cells, the roles of TLRs 2, 3, 4, and 9 have been precisely investigated. Overall, these TLRs, after engagement, led to better CD8+ T cell response, with increased cytokine secretion, proliferation, cytotoxic function and memory T cell differentiation 6.

In human CD8+ T cells, different TLRs have been studied and recently reviewed 6. TLR1/2 agonist has been described to increase the production of IFNγ 7,8, IL2 7, perforine and granzyme B 8 and decrease CXCR4 and CCR7 expression 9. TLR3 has then been shown to increase the production of IFNγ by human effector cytotoxic T lymphocytes (CTLs) derived from human blood 10. TLR4 activation has also been reported to increase IFNγ production by CD8+ T cells 11. TLR5 synthetic agonist significantly increased the proliferation and production of IFNγ, perforine, and granzyme B by CD8+ T cells derived from human cord blood samples 8. Finally, some studies have highlighted the involvement of TLR9 in the downregulation of PD-1 in CTLs, resulting in increased proliferation, cytotoxicity and cytokine production of these cells 12. TLR9 agonist has also been shown to increase degranulation and differentiation of melanoma-specific human CTLs 13. The implications of the other TLRs in human CTL function, in particular TLR8, the dimeric structure of which has been recently elucidated 14, have not yet been fully studied, even though their mRNA expression has already been detected in these cells 2,15. TLR7/8 agonist has first been used as a local treatment in cutaneous square cell carcinoma 16 demonstrating that TLR7/8 engagement on human CD8+ T lymphocytes induced an increased production of granzyme B. However, this study could not demonstrate that this effect was due to a direct engagement of TLR7 or of TLR8 on CD8+ T lymphocytes, or to an indirect effect through APCs or CD4+ T lymphocytes. Later, TLR7 and TLR8 have been studied on purified CTLs, but only in particular contexts. TLR7 engagement has been shown to increase CD69 expression and IFNγ production by CTLs purified from blood samples, but only in HIV-1—infected patients 17. TLR8 engagement has been shown to reverse regulatory CD4+ 18,19, γδ 20,21, and CD8+ 22 T cell function *in vitro* and/or *in vivo*.

In the present study, we investigated TLR8 expression and its direct short-term effect on the function of *in vitro* activated antigen-specific human CTLs derived from healthy donor peripheral blood. However, obtaining *in vitro* a sufficient amount of highly specific CTLs capable of retaining cytotoxic activity *in vivo* remains problematic. Therefore, we used artificial APCs (AAPCs) 23 to overcome the difficulties of generating *in vitro* large quantities of highly efficient anti-tumor CTLs for adoptive cell therapy strategies 24,25. We were particularly interested in tumor antigen-specific CTL functional avidity study, since high avidity CTLs have already been described as more efficacious in adoptive cell therapy 26. We first confirmed by PCR and flow cytometry that CTLs expressed different TLRs, and in particular TLR8, in the intracellular compartment and at the cell surface. We then focused our study on the effect of a direct CTL stimulation through TLR8 engagement on tumor antigen-specific CTL function. MART-1, a major melanoma-associated protein, was used as a model antigen in this study. Antigen-specific T lymphocytes activated by a synthetic TLR8 agonist (3M002, CL075) showed increased cytotoxic activity against MART-1-pulsed target cells. TLR8 engagement led neither to any change in the production levels of cytokines implicated in cytotoxicity nor to a major significant change in CD8 cell surface phenotype, but significantly increased the functional avidity 27–29 of MART-1-specific CTLs for their target cells. These results suggest that TLR8 engagement on human CTLs might be useful in immunotherapy strategies.

## Materials and Methods

### Recruitment of healthy donors

Six healthy donors were recruited based on the expression by flow cytometry of HLA-A2 molecule from *Etablissement Français du Sang* local department (Bois-Guillaume, France). They were informed and had given an oral consent for study, in agreement with IRB recommendations (*Comité de Protection des Personnes Nord-Ouest 1*, Rouen, France). Blood samples were collected and then anonymously studied.

### TLR expression study on CD8+ T cells

Toll-Like Receptor 8 (TLR8) expression was analyzed by RT-PCR on purified CD8+ T cells. Peripheral Blood Mononuclear Cells (PBMCs) were collected by density centrifugation on a lymphocyte separation medium (Eurobio laboratories, Courtaboeuf, France). T cells were isolated by rosetting, using sheep red blood cells (Eurobio Laboratories) and a second density separation protocol. CD8+ T cells in erythrocyte-rosetted positive fraction were purified by positive selection using anti-CD8 immunomagnetic beads and MS or LS columns following the manufacturer's recommendations (Miltenyi Biotec, Paris, France). CD8+ T cells were cultured in RPMI (Invitrogen, Cergy-Pontoise, France) with 10% FCS (Fetal Calf Serum, Invitrogen), stimulated with IL-2 (60 IU/ml; Sigma–Aldrich, Lyon, France) and anti-CD3 (1 µg/ml, Invitrogen) during 72 h at 37°C. Total cytoplasmic RNA was extracted before or after activation using genElute Mammalian Total RNA Kit (Sigma–Aldrich) and reverse transcribed using the Superscript II RNAse H-reverse Transcriptase (Invitrogen) following the manufacturer's recommendations. The primer sequences, based on human TLR sequence and used for PCR, were 5′-CAGAATAGCAGGCGTAACACATCA-3′ and 5′-AATGTCACAGGTGCATTCAAAGGG-3′. Prior to PCR reaction, a denaturation step was achieved at 94°C for 2 min. Then, the following cycle was repeated 30 times: 2 min at 94°C for denaturation, 30 s at 72°C for hybridization and 30 s at 72°C for elongation. A final step at 72°C for 1 min was added to allow a final extension. Actin was used as a positive control of the PCR reaction. Products were separated on 2% agarose gels and stained with ethydium bromide (Sigma–Aldrich).

### Construction of artificial antigen-presenting cells

Vector construction and gene transfer procedure were previously described 23. Briefly, NIH/3T3 mouse fibroblasts (ATCC, Manassas, VA) were cultured in DMEM (Invitrogen) supplemented with 10% Donor Calf Serum (DCS, ThermoFisher Scientific, Illkirch, France) and transduced with gammaretrovirus-derived SFG vectors encoding the HLA-A*0201 heavy chain, β-2-microglobulin, B7.1 (CD80), ICAM-1 (CD54), and LFA-3 (CD58) proteins. Expression of HLA-A*0201 heavy chain, β-2-microglobulin and the co-stimulatory molecules B7.1, ICAM-1, as well as LFA-3 was assessed on the surface of the Artificial Antigen Presenting Cells (AAPCs) by flow cytometry. The genetically engineered AAPCs were also transduced with a dicistronic vector encoding, upstream of the puromycin-N-acetyltransferase open reading frame, the MART-1-derived analogue peptide A27L (M1m, ELAGIGILTV) described as being more immunogenic than the native MART-1 peptide 30. The human CD8α leader sequence was added to the peptidic sequence to target the peptide to the endoplasmic reticulum. Puromycin (Sigma–Aldrich) was added at 5 µg/ml to the medium for 1 week to select the cells expressing the vector-encoded puromycin-N-acetyltransferase.

### Purification of T cells for co-culture

Non-activated T cells were isolated from peripheral blood samples as previously described 23. Briefly, PBMCs from HLA-A*0201+ healthy donors were collected by density centrifugation on a lymphocyte separation medium and T cells were isolated by rosetting, using sheep red blood cells and a second density separation protocol. The T cell-enriched population was collected. Natural Killer (NK) cells, B cells and activated T cells were depleted with a cocktail of mouse monoclonal antibodies directed against CD11b, CD16, and HLA-DP, -DQ, -DR (BD Biosciences, Le Pont de Claix, France) at 1 µg per million cells for 30 min followed by panning on Petri dishes coated with goat anti-mouse IgG (Thermo Scientific, Courtaboeuf, France) as previously described 23. After three washes in PBS (Life Technologies, Saint Aubin, France) with 2% FCS, the T cells were resuspended and maintained in AIM-V medium (Life Technologies) without serum.

### Expansion of antigen-specific CTLs

Co-culture conditions were determined previously 23. Briefly, 10^5^ irradiated AAPCs (25 Gy) resuspended in 500 µl of AIM-V medium with 5% DCS, transduced to express M1m peptide as mentioned above (see “Construction of artificial antigen-presenting cells”) were deposited in the bottom of a well from a 24-well plate one day before the stimulation. 10^6^ total T lymphocytes resuspended in 500 µl AIM-V medium were cultured on the layer of AAPCs for 21 days (CD4+ TL population does not receive any TCR activation signal from our AAPCs but the helper role of this population, even if it is not required, allows an increased amplification of CD8+ TLs and MART-1-specific CTLs [data not shown]).

On day (D) 7 of the co-culture and then, every third day, 20 IU/ml of IL-2 (R&D systems, Lille, France) were added in the wells. Cells were harvested and used in experiments at D21.

### CTL stimulation with TLR8 synthetic agonist

TLR8/7-specific synthetic thiazoloquinoline ligand, CL075 (3M002), was purchased from Invivogen (Toulouse, France), resuspended in sterile endotoxin-free water at a concentration of 1 µg/ml. A wide range of concentration was assessed and a single concentration of 0.4 μM allowing the highest effect on TLR8 pathway without activating TLR7 one 31 was obtained. We have also assessed the effect of TLR8 engagement at different time points, from 4 h (D21) to 2 days after TLR8 engagement (D23). We observed that the effect of TLR8 engagement on CTLs was at its highest 24 h after stimulation (data not shown). Finally, since there is no available quinoline family member molecule which could be used as a relevant control because all quinolines, by nature, could activate different TLRs or TLR-related receptors expressed by CD8+ T cells, we used CL075 solvent (here, sterile endotoxin-free water) as a negative control, as recommended by the manufacturer and as reported in different publications 32,33.

### Purification and amplification of specific T cells

At the end of the co-culture, total cells were resuspended at 10^7^ cells per 100 µl in cold PBS (4°C) supplemented with 0.1% Bovine Serum Albumin (BSA, Sigma–Aldrich) and 2 mM EDTA (VWR, Fontenay-sous-Bois, France). Phyco-Erythrin (PE)-coupled Pro5 MHC class I Pentamer expressing M1m (Pentamer M1m, Proimmune, Oxford, UK) was added in medium (100 ng for 10^7^ cells) for 30 min. Cells were then washed and incubated with anti-PE microbeads (Miltenyi Biotec) for 15 min and finally washed again and resuspended in 500 µl of cold PBS supplemented with 0.1% BSA and 2 mM EDTA. During the different steps of purification, cells were always stored on ice and all reagents were kept at 4°C. Magnetic sorting was realized following manufacturer's protocol. Cells were amplified on AAPCs (5 × 10^5^ T cells on 10^5^ irradiated AAPCs), as described above for 14 days, and then used in both phenotypic and functional studies at D35.

### CTL phenotypic study

For measurement of cell surface markers, CD8+ T cells obtained after one round of stimulation on AAPCs at D21 and M1m-specific cytotoxic T lymphocytes purified by magnetic sorting and reamplified on AAPCs until D35 were stained with antibodies coupled with fluorescein isothiocyanate (FITC), phycoerythrin (PE), peridin chlorophyll protein (PerCP), or allophycocyanin (APC) fluorochromes during 1 h at 4°C. Isotype controls for the different antibodies were used following manufacturers' recommendations. Anti-CD3-PE, anti-CD4-PE, anti-CD8-FITC, anti-CD14-FITC, anti-CD16-FITC, anti-CD19-PE, anti-CD54-PE, anti-CD56-PE, anti-CD58-FITC (Caltag Laboratories, Burlingame, CA), anti-CD2-FITC, anti-CD8-APC, anti-CD18-PE, anti-CD25-PE-Cy7, anti-CD28-PE-Cy7, anti-CD44-PerCP-Cy5.5, anti-CD57-FITC, anti-CD95-FITC, anti-CD122-PE, anti-CD127-FITC,anti-CTLA-4-PE, anti-FasL-PE, anti-PD-1-PerCP-efluor710 (eBiosciences, Paris, France), anti-CD27-APC, anti-CD62L-PE, anti-CCR4-PE, anti-CCR7-PE, anti-BTLA-PE, anti-CLA-FITC (BD Pharmingen), anti-CD69-FITC, anti-CCR10-APC (R&D systems), anti-CD45RA-APC, anti-CD45RO-FITC (ThermoFisher Scientific), and anti-TLR8-PE (Imgenex, San Diego, CA) antibodies were used. Pro5 MHC pentamers were used to stain T lymphocytes specific of the relevant M1m peptide or of a Flu matrix-derived peptide (FMP) as control. The cells were labeled as recommended by the manufacturers. Then, they were washed and resuspended in 400 µl PBS supplemented with 2% FCS. For intracellular staining, the cells were cultured with both M1m-expressing AAPCs and Brefeldin A (20 µg/ml) during 4 h at 37°C and then, fixed and permeabilized using Intraprep kit (Beckman Coulter, Villepinte, France) following the manufacturer's instructions. Anti-CD8-APC (Caltag Laboratories) or anti-CD8-PerCP (BD Pharmingen), anti-TLR8-PE (Imgenex), anti-Granzyme B-FITC, anti-IFNγ-PerCP, anti-TNFα-PE (Immunotech, Marseille, France), and anti-CD107a-APC (BD Pharmingen) antibodies were used. All data were acquired using FACSCalibur and FACSCanto cytometers (BD Biosciences). FlowJo software (Tree Star, Ashland, OR) was used to analyze flow cytometry data.

### CTL cytotoxic and functional avidity studies

Cytotoxicity was measured using a standard Chromium (^51^Cr) release assay. TAP-deficient HLA-A*0201+ T2 cells (ATCC) were resuspended in RPMI medium (500µL), incubated 1 h at room temperature with the peptides (M1m or FMP, synthesized at the laboratory INSERM U413, Rouen, France) at a final concentration of 10 µM and then labeled for 1 h at 37°C with ^51^Cr (PerkinElmer, Courtaboeuf, France). 5000 T2 cells were incubated with total cytotoxic TLs obtained after one round of stimulation on AAPCs at D21 or M1m-specific cytotoxic TLs purified by magnetic sorting and reamplified on AAPCs until D35 at different ratios (40: 1 to 5: 1 or 10: 1 to 1.25: 1, respectively) during 4 h at 37°C. Chromium release was calculated by the formula: ([measured experimental cpm−mean spontaneous cpm]/[mean maximum cpm–mean spontaneous cpm]) × 100.

To study CTL functional avidity, we performed the following test. T2 cells were incubated with different concentrations of MART-1 or FMP peptide, from 1 to 10^−3^ µM. For each peptide concentration, both T lymphocyte (TL) populations defined above (total CTLs or purified M1m+ CTLs) were incubated with T2 target cells at an effector to target ratio of 10: 1 or 2.5: 1, respectively. Chromium release was calculated as explained above.

### Cytokine secretion analysis

IFNγ and TNFα productions were measured in cell-free supernatant of total CTLs obtained after one round of stimulation on AAPCs at D21 using a BIOplex kit (Millipore Corporation, Billerica, MA), following manufacturer's protocol. Data were acquired on a Bio-Plex HTF system (Bio-Rad, Marnes-la-Coquette, France).

### Statistic analysis

Graphpad Prism software (GraphPad Software, Inc., La Jolla, CA) was used to perform *t* tests between the control and CL075 treated groups. *p* values are indicated on graphs. Histograms are represented with standard error of mean (SEM). ns (non significant) was used when *p *> 0.05, * when *p *< 0.05, ** when *p *< 0.01, *** when *p *< 0.001.

## RESULTS

### Human CD8+ T cells express TLR8 in the intracellular compartment and at the cell surface

**Figure 1:**
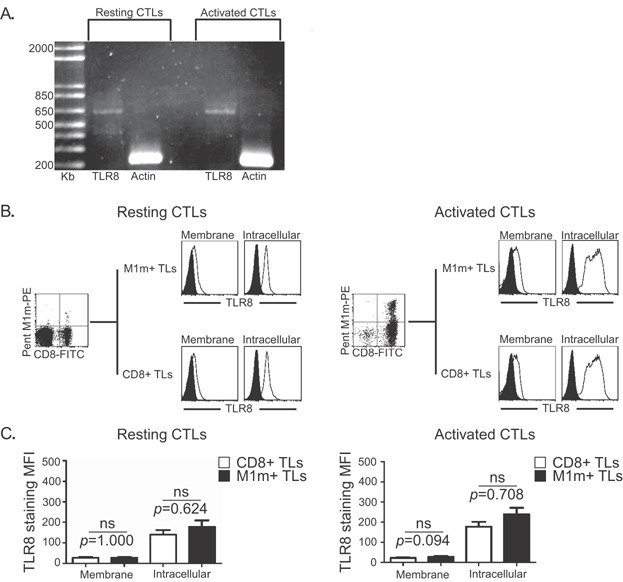
TLR8 expression by CD8+ T lymphocytes. A: Example of TLR8 expression analysis by RT-PCR with (Activated CTLs) or without (Resting CTLs) cell activation by anti-CD3 and IL-2. Actin was used as a positive control of the PCR reaction. The obtained bands are at the expected sizes. B,C: TLR8 expression was evaluated by flow cytometry with PE-conjugated anti-TLR8 antibody at the cell surface (Membrane) and in the intracellular compartment (Intracellular) of MART-1-derived A27L peptide (M1m)-specific T lymphocytes (M1m+ CTLs) and M1m-CD8+ TLs (CD8+ TLs), obtained before (Resting) and after co-culture (Activated) with M1m-expressing artificial APCs. B: Example of TLR8 expression of CD8+ TLs (lower profiles) and M1m+ TLs (upper profiles). White profiles: control isotype. Black profiles: specific staining. C: Mean fluorescence intensity (MFI) of TLR8 staining in CD8+ TLs (white bars) and M1m+ TLs (black bars) Data are represented with standard errors of means. These results have been obtained with six healthy donors, each donor having been studied in three independent co-culture experiments. Statistical tests (*t* tests) have been performed to compare both conditions. Non significant (ns): *p *> 0.05.

After purification from Peripheral Blood Mononuclear Cells (PBMCs) of six Human Leukocyte Antigen (HLA)-A*0201 healthy donors, magnetic bead-sorted cells contained reproducibly more than 98% of CD8+ T lymphocytes (CD8+ TLs, data not shown). These purified CD8+ TLs expressed TLR8 mRNAs independently of the activation state of the cells (Fig. 1A), with inter-individual variations (data not shown). We validated TLR8 mRNA expression by sequencing the amplified PCR product. Flow cytometry analysis confirmed TLR8 protein expression by M1m-specific CD8+ TLs (M1m+ TLs) and CD8+ TLs (Fig. 1B), independently of TL activation state (Fig. 1B) and at a high level in the intracellular compartment and at a lower level at the cell surface. There was no significant difference in TLR8 expression between M1m+ TLs and CD8+ TLs (Fig. 1C), neither in the intracellular compartment (*p* = 0.624 and *p* = 0.708, respectively) nor at the cell surface (*p* = 1.000 and *p* = 0.094, respectively).

### TLR8 engagement on CTLs, 24 h before their study, does not modify antigen-specific CTL percentage and TCR expression level

**Figure 2:**
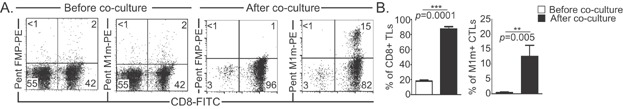
Activation of MART-1-specific T lymphocytes with AAPC system. A: Example of MART-1-specific CTLs obtained after one round of stimulation on AAPCs at D21 and assessed using MART-1 Pentamer (Pent M1m) staining. FMP Pentamer (Pent FMP) was used as control. Relative percentages are indicated in each quadrant. B: Mean percentages of CD8+ TLs (left panel) and MART-1-specific cytotoxic TLs (M1-CTLs, right panel) before (white histogram) and after (black histogram) co-culture with AAPCs. Data are represented with standard errors of means. These graphs represent the results obtained with six healthy donors, each donor having been studied in three independent co-culture experiments. Statistical tests (*t* tests) have been performed to compare both conditions. **p < 0.01.

Non-activated T cells with more than 98% purity (data not shown) were obtained from the peripheral blood of HLA-A*0201 healthy donors and co-cultured with Artificial Antigen Presenting Cells (AAPCs) expressing an HLA-A*0201-restricted MART-1-derived analogue peptide (AAPC^M1m^). Depending on the donor, 3–27% of cells obtained *in vitro* after co-culture were MART-1-specific CTLs (Fig. 2A).

**Figure 3:**
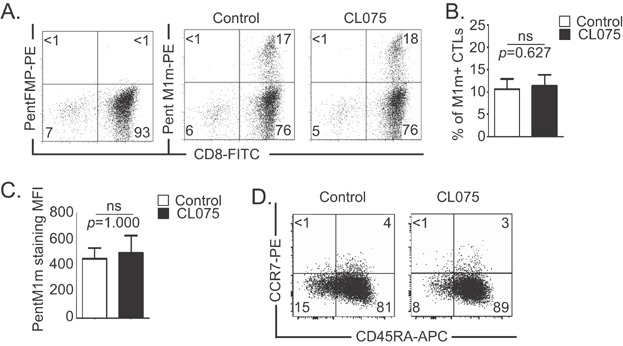
TLR8 pathway stimulation does not increase the percentage of MART-1-specific CTLs or TCR expression level. A,B,C,D: MART-1-specific CTLs were obtained after one round of stimulation on M1m-expressing AAPCs at D21. TLR8 pathway stimulation was achieved by adding TLR8 synthetic agonist (CL075) 24 h before the end of the co-culture. A: Example of percentage of M1m+ CTLs assessed using MART-1 Pentamer (PentM1m) staining. FMP Pentamer (PentFMP) was used as control. Relative percentages are indicated in each quadrant. B: Mean percentages of M1m+ CTLs with (black histogram) or without (white histogram) TLR8 pathway stimulation. C: Mean fluorescence intensities (MFI) of Pentamer MART-1 staining on CTLs with (black histogram) or without (white histogram) TLR8 pathway stimulation. B. C. Data are represented with standard errors of means. These graphs represent the results obtained with six healthy donors, each donor having been studied in three independent co-culture experiments. Statistical tests (*t* tests) have been performed to compare both conditions. Non significant (ns): *p *> 0.05. D: Example of CD45RA and CCR7 double staining on total CTLs. Relative percentages are indicated in each quadrant.

The same experiment was performed with six healthy donors, revealing that both CD8+ TL and MART-1-specific CTL populations were very significantly amplified (*p* = 0.0001 and *p* = 0.005, respectively) in our co-culture conditions (Fig. 2B). We found that activation with TLR8 synthetic agonist (CL075, 3M002) 24 h before the end of the co-culture triggered no change of CD8+ T cell population in terms of absolute number (data not shown), of MART-1-specific CTL percentage (Fig. 3A and B, *p* = 0.627), or of MART-1-specific TCR expression level (Fig. 3C, *p* = 1.000) according to the mean fluorescence intensity (MFI) after MART-1 pentamer staining. However, we observed that, overall, the percentage of CD45RA+ CCR7-TLs was slightly increased after TLR8 engagement (Fig. 3D).

### TLR8 activation of CTLs increases cytotoxicity and antigen-specific functional avidity

**Figure 4:**
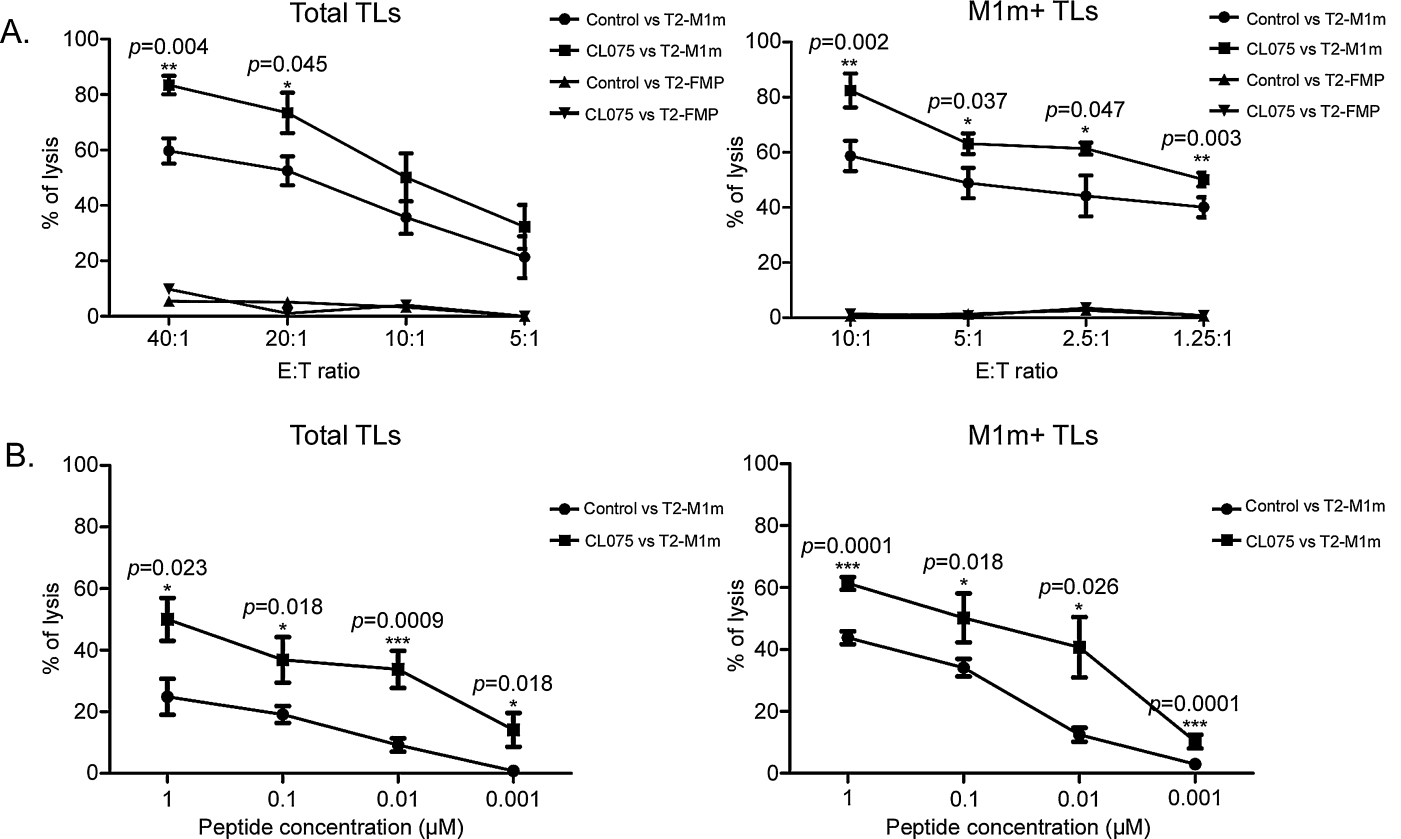
TLR8 pathway stimulation increases specific cytotoxicity and functional avidity of CTLs. A. B. Total T lymphocytes (Total TLs, left panels) were obtained after one round of stimulation on M1m-expressing AAPCs at D21. Purified MART-1-specific cytotoxic TLs (M1m+ CTLs, right panels) were reamplified on M1m-expressing AAPCs until D35. TLR8 pathway stimulation was achieved by adding TLR8 synthetic agonist (CL075) 24 h before the end of the co-culture. Data are represented with standard errors of means. These graphs represent the results obtained with six healthy donors, each donor having been studied in three independent co-culture experiments. Statistical tests (*t* tests) have been performed to compare both conditions. When lower than 0.05, *p* values are indicated. *p < 0.05, **p < 0.01, ***p < 0.001. A: Cytotoxic activity of total CTLs and M1m+ CTLs was assessed in standard 4-h ^51^Cr release assays. Target cells were T2 cells pulsed with MART-1-derived peptide (T2-M1m) or Flu Matrix Protein-derived peptide (T2-FMP) as control at a peptide concentration of 10 μM. B: Cytotoxic specific activity of total CTLs and M1m+ CTLs with (squares) or without (circles) TLR8 pathway stimulation was assessed in standard 4-h ^51^Cr release assays at the effector to target ratio of 10:1 and 2.5:1, respectively. Target cells were T2 cells pulsed with various concentrations (from 1 to 10^−3^ µM) of MART-1-derived peptide (T2-M1m) or Flu Matrix Protein-derived peptide (T2-FMP) as control for non-specific lysis. The latter was always lower than 10% and was subtracted to calculate the specific lysis displayed on the graphs.

Total T cells obtained after co-culture with AAPC^M1m^ and purified M1m+ TLs were used in cytotoxic assays to investigate cytolytic function of MART-1-specific CTLs. We observed that CTLs obtained *in vitro* were able to specifically kill target cells that presented MART-1-derived peptide in every culture we performed (Fig. 4A) with purified M1m+ TLs displaying higher specific cytotoxic capacities (Fig. 4A right panel). Among the six tested HLA-A*0201 healthy donors, we found significant increased cytotoxicity (from 10% to 20%) after addition of TLR8 synthetic agonist, at all tested ratios for purified M1m+ TLs (*p *< 0.05, Fig. 4A right panel) and at least for the highest ratios for total TLs (40:1 and 20:1, *p* = 0.004 and *p* = 0.045, respectively, Fig. 4A left panel). The two donors with the highest responses after TLR8 engagement had the lowest percentages of MART-1-specific CTLs obtained after co-culture (3% and 4%, data not shown). On the contrary, the two donors that showed the lowest increases in cytotoxic response after TLR8 stimulation had the highest percentages of MART-1-specific CTLs (more than 20%, data not shown).

We further investigated the mechanism by which TLR8 pathway activation could modulate the activity of peptide-specific CTLs. We designed a functional avidity test based on our standard chromium assay. CTL populations obtained from six healthy donors, containing at least 3% of MART-1-specific CTLs after one co-culture and more than 95% after M1m+ TL purification, incubated with TLR8 synthetic agonist, were able to specifically kill T2 cells loaded with a wide range of peptide concentrations, from 1 to 10^−3^ µM, with still more than 10% of specific lysis at the lowest concentration (Fig. 4B). At each tested concentration, CTLs treated with TLR8 synthetic agonist presented a significantly higher functional avidity for target cells than control CTLs for both conditions (*p *< 0.05, Fig. 4B).

### TLR8 activation of CTLs does not increase IFNγ, Granzyme B and TNFα productions but slightly increases CD107a expression

To elucidate how TLR8 synthetic agonist can increase the cytotoxicity and functional avidity of CTLs expanded *in vitro*, we studied the production of different factors implicated in cytotoxicity by flow cytometry.

**Figure 5:**
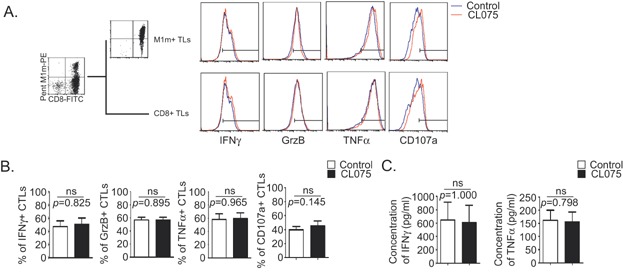
TLR8 pathway stimulation does not increase IFNγ, Granzyme B or TNFα production but slightly increases degranulation by CD8+ T lymphocytes. A,B: Total T lymphocytes (Total TLs, left panels) were obtained after one round of stimulation on M1m-expressing AAPCs at D21. Purified MART-1-specific cytotoxic TLs (M1m+ CTLs, right panels) were re-amplified on M1m expressing AAPCs until D35. TLR8 pathway stimulation was achieved by adding TLR8 synthetic agonist (CL075) 24 hours before the end of the co-culture. A: Example of IFNγ, Granzyme B, TNFα, and CD107a staining in total CTLs (lower panels) and in M1m+ CTLs (upper panels), with (red curve) or without (blue curve) TLR8 pathway stimulation. Populations were activated for 4 h with M1m-expressing AAPCs while their secretion was blocked using Brefeldin A before staining. Bars on histograms were placed according to isotype control staining. B: Mean percentages of IFNγ, Granzyme B, TNFα, and CD107a-specific CTLs in total CTLs were assessed, with (black histograms) or without (white histograms) TLR8 pathway stimulation. C: IFNγ and TNFα concentrations were measured in the supernatants of total CTLs with (black histograms) or without (white histograms) TLR8 pathway stimulation. B,C: Data are represented with standard errors of means. These graphs represent the results obtained with six healthy donors, each donor having been studied in three independent co-culture experiments. Statistical tests (*t* tests) have been performed to compare both conditions. Non significant (ns): *p *> 0.05.

In term of mean fluorescent intensities, we found that there was no significant difference between control and TLR8-stimulated populations for IFNγ, Granzyme B and TNFα productions in purified M1m+ TLs at D35 (*p *> 0.05, Fig. 5A upper panels) and CD8+ TLs at D21 (*p *> 0.05, Fig. 5A lower panels) under our co-culture conditions. However, TLR8 engagement had a tendency to increase the mean fluorescence intensities of CD107a staining (Fig. 5A).

In term of percentage of positive cells, we found no significant differences between control and TLR8-stimulated populations for the different molecules measured (*p *> 0.05, Fig. 5B) with a tendency to have an increased CD107a+ CTL population after TLR8 engagement (Fig. 5B right bars).

Measurement of IFNγ and TNFα released in cell supernatants of total TLs at D21 showed no significant variation with or without TLR synthetic agonist, in our system (*p *> 0.05, Fig. 5C).

### TLR8 engagement on CTLs does not lead to major cell surface phenotypic changes but slightly increases CD45RA, CD62L, and CCR10 expression, and slightly decreases CCR7, CTLA4, and BTLA expression

**Figure 6:**
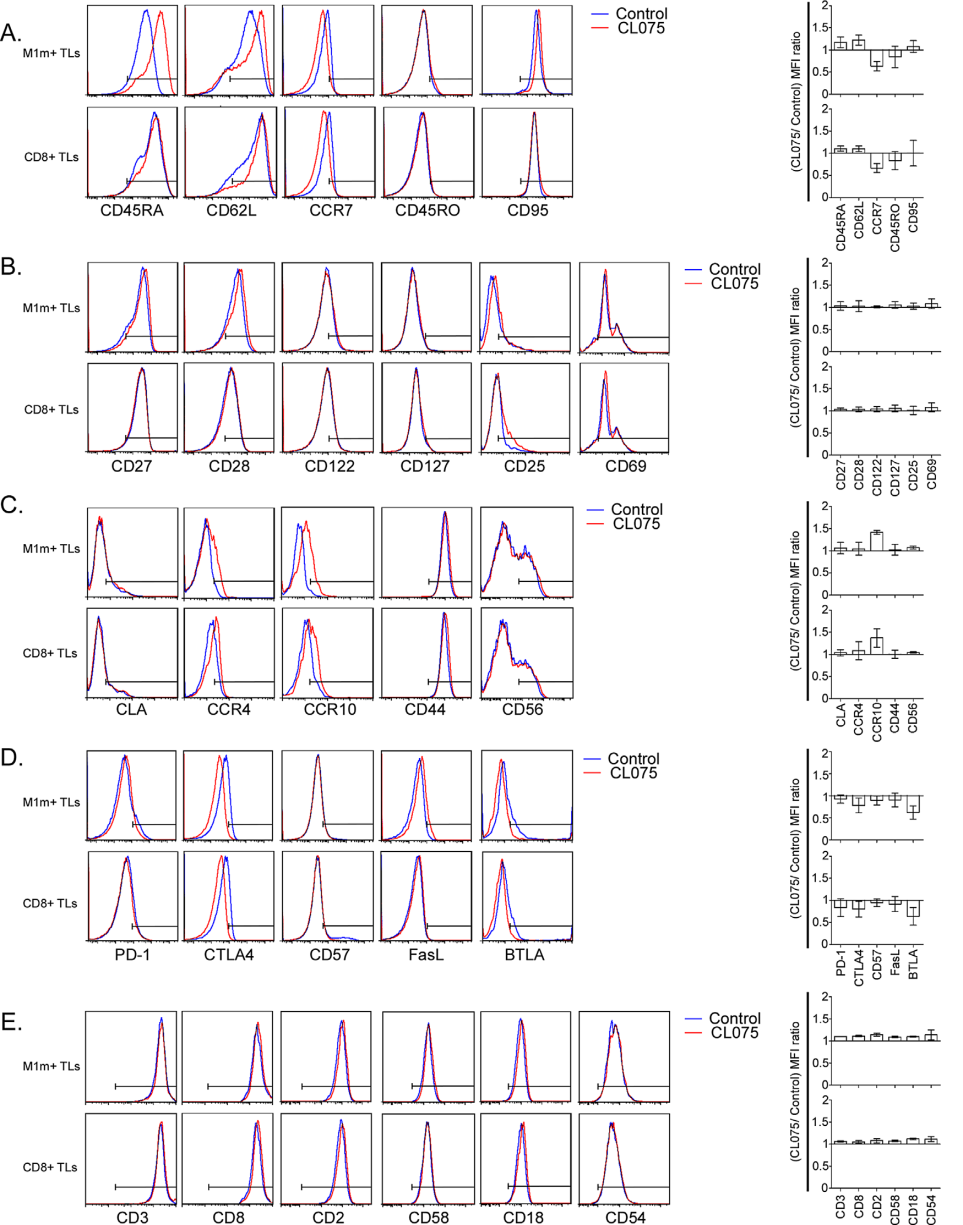
TLR8 engagement on CTLs slightly increases CD45RA, CD62L and CCR10 expression, and slightly decreases CCR7, CTLA4, and BTLA expression. A,B,C,D,E: Left panels: representative staining examples of total cytotoxic T lymphocytes obtained after one round of stimulation on AAPCs at D21 (CD8+ CTLs, lower panels) and of M1m-specific T lymphocytes purified by magnetic sorting and re-amplified on AAPCs until D35 (M1m+ CTLs, upper panels). TLR8 pathway stimulation was achieved by adding TLR8 synthetic agonist (CL075) 24 h before the end of the co-culture. Bars on histograms were placed according to isotype control staining. Right panels: ratios of mean fluorescence intensities (MFI) of the different markers studied on CD8+ CTLs (lower histograms) and on M1m+ CTLs (upper histograms). Ratios were calculated using the formula: MFI of the corresponding marker in TLR8 pathway-stimulated TLs/MFI of the corresponding marker in the control TLs. These graphs represent the results obtained with six healthy donors, each donor having been studied in three independent co-culture experiments. Cell surface markers are organized as following: A. Differentiation state markers. B. Activation state markers. C. Homing markers. D. Functional inhibition markers. E. Cell interaction and functional avidity markers.

To understand the mechanism which allows higher cytotoxicity and functional avidity after TLR8 stimulation, we studied numerous molecules expressed on CTLs. With the corresponding antibodies, we studied different molecules reflecting: (i) differentiation state (CD45RA, CD45RO, CD95); (ii) lymph node (CD62L, CCR7), skin (CLA, CCR4, CCR10) or inflammated tissue (CD44, CD56) homing capacity; (iii) activation state (CD27, CD28, CD122, CD127, CD25, CD69); (iv) T cell functional inhibition (PD-1, CTLA4, CD57, FasL, and BTLA); and (v) cell interactions and TL functional avidity (CD3, CD8, CD2, CD58, CD18, CD54). There was not any noticeable change of expression in any donor CTLs for CD45RO, CD95, CD27, CD28, CD122, CD127, CD25, CD69, CLA, CCR4, CD44, CD56, PD-1, CD57, FasL, CD3, CD8, CD2, CD58, CD18, and CD54 (Fig. 6). No significant major changes were found in the mean fluorescence intensities and percentages of positive cells (*p *> 0.05, Fig. 6 and data not shown) for any of the different markers we studied. Nevertheless, we found that TLR8 engagement had a tendency to increase the mean fluorescence intensities and the percentages of positive cells for CD45RA, CD62L, and CCR10 in the different donors we studied. Conversely, we found in these donors that TLR8 engagement had a tendency to decrease the mean fluorescence intensities and the percentages of positive cells for CCR7, CTLA4, and BTLA (Fig. 6 and data not shown).

## Discussion

In this study, we found that Toll-like Receptor 8 (TLR8) was expressed in the intracellular compartment as well as at the membrane, although at a low level, for both CD8+ T lymphocytes (CD8+ TLs) and M1m-specific CD8+ T lymphocytes (M1m+ TLs), independently of their activation state. We assessed a wide range of concentrations of CL075 (3M002), a synthetic TLR8/7 agonist (data not shown), and a single concentration allowing the highest TLR8 effect without triggering TLR7 pathway was chosen. The addition of TLR8 synthetic agonist 24 h before a cytotoxic assay using both total TLs and purified M1m+ TLs obtained by co-culture with Artificial Antigen Presenting Cells (AAPCs) led to a significant increase in peptide specific cytolysis. This effect could only be related to the direct action of the agonist on the CTLs and not on the AAPCs or on the CD4+ T cells. When the agonist was added to total TL or M1m+ TL co-cultures, the AAPCs had already been totally destroyed by the activated CTLs, a phenomenon which had occurred around the 7th day of co-culture for total TLs and the 1st day of reamplification after purification for M1m+ TLs, 2 weeks before TLR8 engagement. CD4+ T cell potential role was ruled out in cultures where CD8+ TLs represented more than 98% of obtained cells at the end of the first co-culture or after M1m+ CTL purification, with a strong TLR8 synthetic agonist effect. Since for some donors a small population of CD4+ T cells survived throughout the first co-culture, nevertheless, we were interested in the potential role it could play in the effect we observed. We found that there was neither a positive nor a negative correlation between the percentage of CD4+ T cells and the effect on CTL cytotoxicity, arguing against a significant role of CD4+ T cell population, even in these cultures.

In all cultures, TLR8 pathway activation led to higher cytotoxicity without any change neither in percentage and absolute number of M1m+ TLs nor in production of cytotoxicity-related factors. However, we found that the mean fluorescence intensity of CD107a staining was slightly increased after TLR8 engagement. This observation highlights the fact that TLR8 engagement slightly favours CTL degranulation in our culture conditions. Another TLR7/8 synthetic ligand has already been used on Peripheral Blood Mononuclear Cells (PBMCs) 34 and on cutaneous square cell carcinoma in humans 16. These two studies reported interesting findings such as increased production of perforine and granzyme B in CD8+ T cells. Even if we were not able to reliably measure perforin expression, we did not find any correlation between the effect of TLR8 stimulation and a significant increase in the production of different molecules associated with cytotoxicity: IFNγ, Granzyme B and TNFα. Contaminant cells in human CD4+ T cell fraction have been shown to add some co-stimulatory signals which could induce cytokine production by these CD4+ T cells 35. Since in our experiments, we had pure populations of human T lymphocytes, with more than 98% of CTLs at the end of some co-cultures and always with more than 95% of M1m+ TLs after M1m+ TL purification, the absence of contaminant cells could explain the differences observed with these studies.

Another explanation could be that we used concentrations that allowed only TLR8 engagement 31, when in the aforementioned publications, both TLR7 and TLR8 pathways were probably stimulated. However, these two studies provide interesting perspectives on TLR7/8 engagement in cancer treatment. In addition, TLR8 engagement has been shown to block both regulatory CD4+ and γδ TL function 18,20 by inhibiting their capacity to induce a telomere-independent senescence in effector TLs 19,21. Conversely, different studies have described pro-tumoral effects when TLR pathways were activated in tumor cells, including TLR8 6, arguing against the systemic or local use of TLR8 synthetic agonist in cancer immunotherapy. Altogether, these data and ours highlight, on the contrary, the interest of using TLR8 synthetic agonist *in vitro* in cancer adoptive cell therapy.

The observation of increased T cell cytotoxicity without correlation with a higher production of cytotoxic factors led us to the following hypothesis: in humans, TLR8 activation might play a role by decreasing the level of stimulation that a T cell requires to become activated and to kill its target cells rather than by increasing the cytotoxic potential of CTLs directly through cytotoxic molecule higher expression levels. We investigated this hypothesis in six healthy donors. We found that the incubation of CTLs with TLR8 synthetic agonist induced an increased functional avidity, as defined by different groups 27–29, which were able to kill cells incubated with 10-fold less peptide than the control population for both total TLs and purified M1m+ TLs. We hypothesized that this effect could be due to an increased expression of the T cell receptor (TCR) and/or of different signal molecules implicated in CTL activation and cytotoxicity after TCR recognition. In control and TLR8-activated CTLs, we compared the MFI of Pentamer MART-1 staining and the expression of different molecules reflecting the functional state of CTLs. We found that TLR8 engagement did not significantly increase the MFI of Pentamer MART-1 staining, indicating that TLR8 engagement on CTLs did not increase the number or the affinity of TCR complexes at the cell surface. Moreover, TLR8 engagement did not increase the expression levels of CD3, CD8, CD2 and its ligand CD58, and LFA-1 (assessed using its β chain CD18) and its ligand CD54, all molecules potentially playing a role in TL functional avidity 36,37.

Even for all the other different markers we studied, there was no major significant change in the cell surface phenotype of TLR8-stimulated CTLs. However, we found that, on TLR8-stimulated CTLs, the expression of BTLA and CTLA4, two inhibitors of T cell function, slightly decreased, and the expression of CD45RA and CD62L, two markers of non-differentiated CTLs described as being of great interest for adoptive cell therapy 38, slightly increased. Likewise, lymph node homing factor CCR7 expression slightly decreased while skin homing factor CCR10 expression slightly increased. All these moderate differences could nevertheless play a role in the effect of TLR8 engagement we observed on CTL function.

We could observe that almost all TLs obtained in this study were effector memory cells (CD45RA+/− CCR7−). TLR8 engagement triggered a slight change from CD45RA− to CD45RA+ effector memory TLs. However, since almost no expression of different markers of exhaustion and/or of terminal effector memory differentiation (PD-1, CTLA4, CD57, FasL, and BTLA) was found in the obtained population, we could not conclude on a possible role of TLR8 engagement in the acquisition of a more terminally differentiated phenotype by these effector memory TLs (CD45RA+ CCR7− CD127− CD57+).

Altogether, this phenotypic study suggests that TLR8 engagement on CTLs could lead to the obtention of functionally more relevant CTLs for melanoma immunotherapy, with a particular property to home to the skin.

We could not precisely determine the molecular intracellular mechanisms involved in the effects of TLR8 engagement on CTLs. Interestingly, a recent paper showed that TLR9 engagement led to the hyperphosphorylation of TCR complexes in CD4+ T cells, with increased TCR signaling 39. We did not investigate this hypothesis but it remains an interesting issue for further studies.

To evaluate the long-term effects of TLR8 signaling on CTLs and to confirm the interests of our *in vitro* results, TLR8 engagement could be studied *in vivo* in different mouse models. NOD/SCID immunodeficient mice could be used to investigate TLR8 agonist-treated specific CTL effects on tumor regression after injection of human melanoma cells. Since different studies have demonstrated that mouse TLR8 is a functional receptor, as reviewed recently 40, MT-ret/AAD C57BL/6 humanized HLA-A*0201 transgenic mice 41, expressing the HLA-A*0201 extracellular domains fused to the H2-D transmembrane domain and spontaneously developing melanoma, could even more accurately allow the investigation of TLR8 agonist-treated specific CTL effects on melanoma development control.

Finally, we clearly showed in this study that TLR8 engagement increased melanoma peptide-specific CTL cytotoxicity and functional avidity *in vitro*, and that these CTLs could display a more suitable phenotype for immunotherapy. Therefore, TLR8 synthetic agonist could be useful as an adjuvant for the adoptive transfer of T lymphocytes.
